# Adolescent PR3-ANCA-positive hypertrophic pachymeningitis: A case report and review of the literature

**DOI:** 10.1097/MD.0000000000010521

**Published:** 2018-04-27

**Authors:** Kotaro Matsumoto, Mitsuhiro Akiyama, Nobuhiko Kajio, Kotaro Otomo, Kazuko Suzuki, Naoshi Nishina, Kento Kasuya, Naoki Oishi, Kaori Kameyama, Tsutomu Takeuchi

**Affiliations:** a Division of Rheumatology, Department of Internal Medicine; b Department of Otolaryngology, Head and Neck Surgery; c Department of Diagnostic Pathology, Keio University School of Medicine, Shinjuku-ku, Tokyo, Japan.

**Keywords:** adolescence, hypertrophic pachymeningitis (HP), proteinase-3 anti-neutrophil cytoplasmic antibody (PR3-ANCA)

## Abstract

**Rationale:**

Hypertrophic pachymeningitis (HP) is an uncommon, life-threatening disease that is seen in elderly patients with antineutrophil cytoplasmic antibody (ANCA) positivity. Proteinase-3 (PR3)-ANCA-positive HP has not been reported in adolescents. Here, we report the first case of adolescent PR3-ANCA-positive HP successfully treated with immunosuppressive therapy.

**Patient concerns:**

A 14-year-old female presented with fullness and pain in her right ear unresponsive to antibiotics. Laboratory tests showed an elevated C-reactive protein and PR3-ANCA positivity. Computed tomography and magnetic resonance imaging revealed mastoiditis in the right temporal bone. Surgical biopsy revealed severe fibrosis and prominent inflammatory-cell infiltration. She received prednisolone and methotrexate therapy, and then underwent a right mastoidectomy. Five months later, she developed headache, dysarthria, and multiple cranial nerve palsies. Further imaging revealed enhancement and thickening of the right hemispheric dura.

**Diagnosis:**

PR3-ANCA-positive HP.

**Interventions:**

She was successfully treated with steroid pulse therapy for 3 days, followed by high doses of prednisolone and intravenous cyclophosphamide.

**Outcome:**

The treatment resulted in significant improvement of her symptoms, laboratory data, and radiologic findings.

**Lessons:**

PR3-ANCA-positive HP can present not only in the elderly, but also in adolescence, and prompt diagnosis and treatment with immunosuppressive therapy is vital.

## 1 Introduction

Hypertrophic pachymeningitis (HP) is a rare, severe condition involving focal or diffuse thickening of the dura mater.^[1]^ HP can be classified as idiopathic or secondary (due to autoimmune diseases, IgG4-related disease, infections, or malignancies).^[2,3]^ The classification of HP is clinically important, because the treatment approach is based on its etiology, but it is always challenging due to the difficulty of accessing the central nerve systems for biopsy. Unfortunately, if biopsy is feasible, histological examination frequently results in nonspecific findings.^[4]^ Recent studies have revealed that the presence of antineutrophil cytoplasmic antibody (ANCA) is a useful indicator of ANCA-associated vasculitis as the cause.^[5]^ ANCA-positive HP frequently presents as a focal lesion without any other organ involvement in middle-aged to elderly patients.^[5]^

Here, we report the first case of adolescent PR3-ANCA-positive HP. The early recognition and diagnosis of adolescent ANCA-positive HP is important for early appropriate treatment initiation to prevent irreversible organ damage and poor prognosis.

## 2 Presenting concerns

A 14-year-old female with no past history or family history of autoimmune disorders noticed fullness and pain in her right ear in October, 2016. No other symptoms, such as fever or weight loss, were present. Suspected otitis media was treated with ceftriaxone and levofloxacin, but her symptoms did not improve.

## 3 Clinical findings

**Figure 1:**
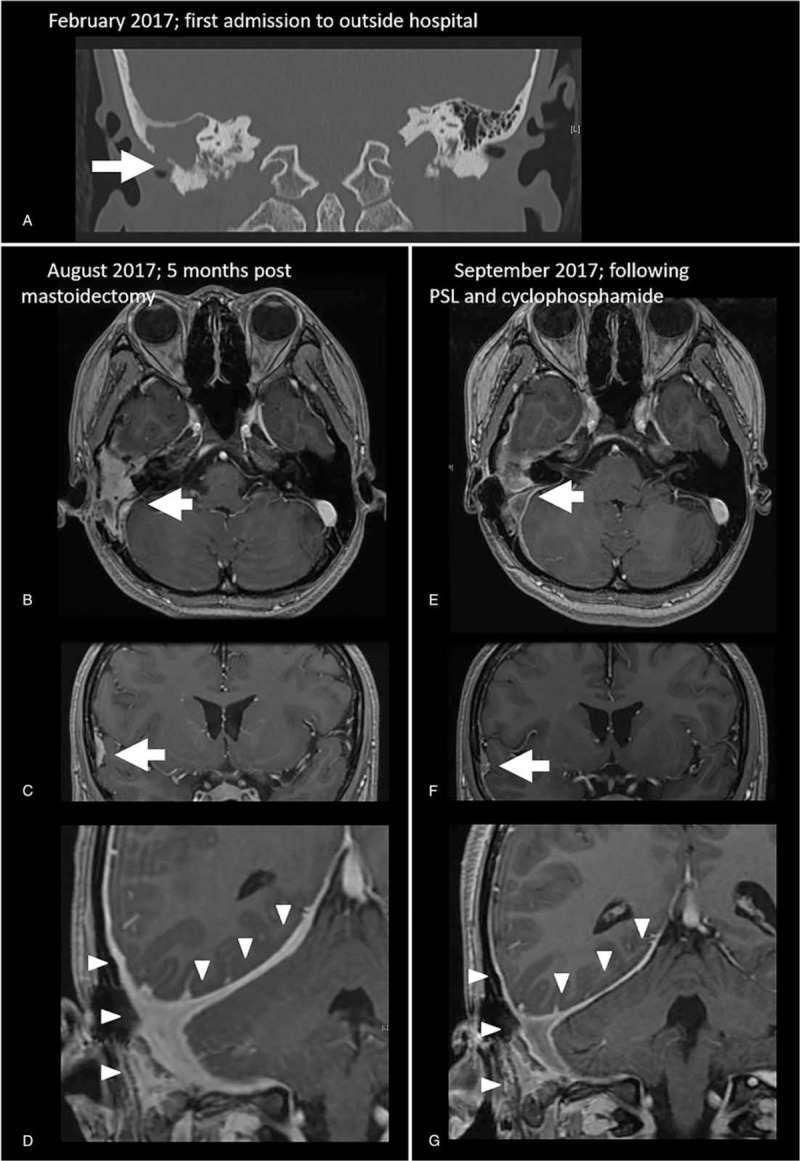
Image findings of mastoiditis and hypertrophic pachymeningitis. Computed tomography (CT) (A) and gadolinium-enhanced magnetic resonance imaging (MRI) (B) revealed right-sided mastoiditis (arrow). MRI revealed extensive hypertrophy of the right side dura mater (C, D) (arrow). MRI findings 1 month after the initiation of induction therapy revealed improvement of mastoiditis (E) and dura mater thickening (F, G).

**Figure 2:**
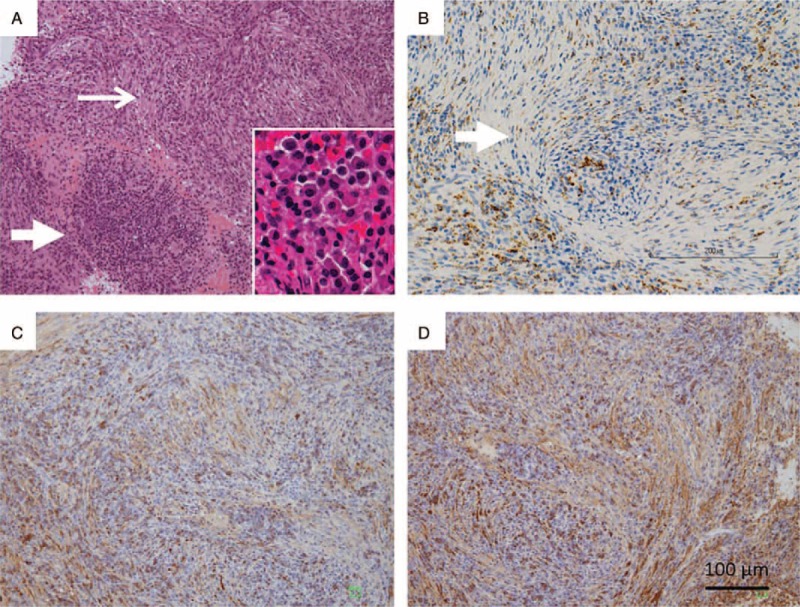
Histological findings of mastoiditis. Hematoxylin-eosin staining (A) (100×) shows prominent inflammation (thick arrow) and severe fibrosis (thin arrow) without granulomatous inflammation or necrotizing vasculitis. The inflammatory cells (inset, 400×) included lymphocytes, plasma cells, and neutrophils. CD68-staining (B) (100×) shows infiltration of macrophages (brown) along with fibrosis (arrow). κ chain (C) and λ chain (D) staining (100×) show no monoclonal proliferation of immunoglobulin light chain.

She visited a pediatrician in another hospital in February, 2017, and laboratory tests showed an elevated C-reactive protein (CRP) (3.2 mg/dL, normal <0.35 mg/dL) and PR3-ANCA (26 U/mL, normal <2 U/mL). Serum levels of IgG (1560 mg/dL, normal range 870–1700 mg/dL) and IgG4 (75 mg/dL, normal range 4.8–105 mg/dL) were normal. Other blood tests, including blood cell count, serum electrolyte levels, liver enzyme levels, and blood glucose, were all within normal range. Urinalysis showed no abnormal findings. Computed tomography (CT) (Fig. 1A) and gadolinium-enhanced magnetic resonance imaging (MRI) (Fig. 1B) revealed mastoiditis involving the right temporal bone. A biopsy revealed prominent inflammation and severe fibrosis without granulomatous inflammation or necrotizing vasculitis. The inflammatory cells included lymphocytes, plasma cells, and neutrophils (Fig. 2A). Immunohistochemistry testing showed the presence of CD68-positive macrophages along with the fibrosis (Fig. 2B). Staining for κ and λ chains showed no restriction of immunoglobulin light chains (Fig. 2C and D). She was suspected of having inflammatory mastoiditis and received 30 mg/d of prednisolone (PSL) from March, 2017.

## 4 Diagnostic focus and assessment

Because of inadequate therapeutic response, the patient was referred to our hospital for further work-up in April, 2017. She underwent a right mastoidectomy, and her symptoms resolved. At the outpatient clinic, 8 mg/wk of methotrexate was added during tapering of the PSL. However, 5 months after the initial treatment, she noted the onset of headache and dysarthria, and was readmitted to our hospital.

Physical examination revealed orbicularis oculi muscle paralysis, sensory deafness, and vocal cord paralysis on the right side, with leftward uvular deviation, which suggested paralysis of the right cranial nerves VII to X. Laboratory tests revealed elevated white blood cell count (13.9 × 10^9^/L, normal range 3.5–8.5 × 10^9^/L), CRP (4.6 mg/dL), and erythrocyte sedimentation rate (111 mm/h, normal <15 mm/h) with persistent elevation of PR3-ANCA (26 U/mL). The levels of hemoglobin (10.7 g/dL, normal range 13.5–17 g/dL) and albumin (3.7 g/dL, normal range 3.9–5.2 g/dL) were low. Other blood tests, including liver enzyme levels, renal function, and blood glucose levels, were within the normal ranges. Antinuclear antibody, rheumatoid factor, anticyclic citrullinated peptide antibody, and myeloperoxidase (MPO)-ANCA tests were all negative. Blood cultures did not identify any pathogens. β-D glucan and interferon-gamma release assays were negative. Urinalysis showed no abnormal findings. MRI revealed extensive hypertrophy of the right hemispheric dura mater (Fig. 1C and D). A chest CT and urinalysis were normal. She was diagnosed with PR3-ANCA-positive HP.

## 5 Therapeutic focus and assessment

She was treated with 1 g/d of intravenous methylprednisolone for 3 days, followed by 50 mg/d (1 mg/kg/d) of oral PSL and 750 mg (15 mg/kg) of intravenous cyclophosphamide every 4 weeks. All clinical manifestations improved rapidly. CRP and PR3-ANCA decreased to normal levels and the thickened dura mater markedly improved after 1 month (Fig. 1E–G).

### 5.1 Patient consent

Written informed consent for treatment and for publication of this case report has been obtained from the patient and parents.

## 6 Discussion

We report the first case of adolescent PR3-ANCA-positive HP. The patient was successfully treated with immunosuppressive therapy including PSL and cyclophosphamide.

Hypertrophic pachymeningitis is an uncommon and severe disease, characterized by thickening of the dura mater.^[1]^ Delay of diagnosis and delayed initiation of appropriate treatment may result in irreversible organ damage and poor prognosis.^[6]^ A recent epidemiological survey of adult-onset HP from Japan reported that the crude HP prevalence was 0.949 per 100,000 individuals, of which 34% were ANCA-positive.^[7]^ Among those cases, 69% were positive for MPO-ANCA and 31% were positive for PR3-ANCA. The epidemiology of adolescent ANCA-positive HP is still unclear due to its extreme rarity. We reviewed all cases of ANCA-positive HP with disease onset <18 years published between 1978 and 2017.^[8–25]^ As shown in Table 1, there were only 4 cases of adolescent ANCA-positive HP including our present case, and the other 3 cases were positive for MPO-ANCA. The age ranged from 5 to 15 years, and all patients were female. The ethnicity was South American in 3 cases (75%) and Japanese in the present case (25%). The initial symptoms associated with HP were headache (100%), proptosis (75%), hearing loss (25%), dysarthria (25%), and seizures (25%). Organ involvement of all cases was limited to ear, nose, and throat lesions. All cases showed elevation of CRP levels (range 1.2–4.6 mg/dL) or of erythrocyte sedimentation rates (range 30–110 mm/h). Hypertrophy of the dura mater was detected by imaging in all cases. The distribution of HP was diffuse in the present case (25%) and patchy in the other 3 cases (75%). Histological examination was not performed in all cases. All cases were successfully treated with a combination of PSL and cyclophosphamide. Long-term prognosis is unclear, and further accumulation of the cases is required.

**Table 1:**
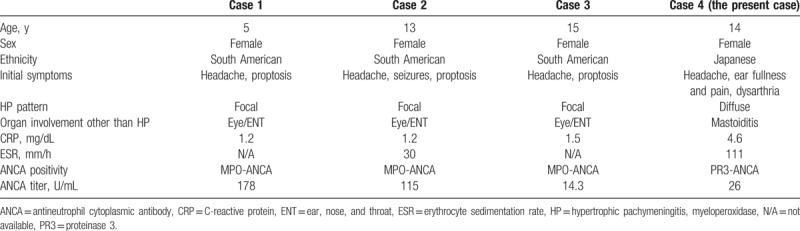
Literature review of ANCA-positive HP with onset <18 years.

Hypertrophic pachymeningitis can be classified based on its etiology, including idiopathic or secondary HP (autoimmune diseases, IgG4-related disease, infections, and malignancies).^[2,3]^ Recent studies have shown that ANCA-positive HP is associated with the limited form of granulomatosis with polyangiitis (GPA).^[5]^ In a previous study, 80% of ANCA-positive HP patients were classified as having the limited form of GPA based on Watts algorithm.^[26]^ Histological examination of HP or upper airway lesions is usually not useful in the diagnosis of GPA due to the usually nonspecific findings such as fibrosis and infiltration of inflammatory cells without granulomatous inflammation or necrotizing vasculitis,^[27,28]^ but it is effective in excluding malignancies and infections. In line with those studies, biopsied specimens of our present case showed nonspecific findings. HP progresses and results in irreversible organ damage when left untreated,^[6]^ but the early initiation of combination therapy with high-dose glucocorticoids and cyclophosphamide prevents this.^[24]^ Thus, when clinicians encounter HP, the limited form of GPA should be considered, and testing for MPO and PR3-ANCA should be performed.

The pathogenesis of ANCA-positive HP remains unclear. Histological findings in the affected tissues of ANCA-positive HP from the previous cases showed severe fibrosis and increased numbers of neutrophils, T cells, plasma cells, and macrophages,^[27]^ similar to our case. Ectopic germinal center formation including B-cell clusters with follicular dendritic cells has also been reported in the lesions of ANCA-positive HP.^[5]^ Thus, the network of T cells, B cells, neutrophils, and macrophages may play an important role in initiating, maintaining, and localizing immune responses, which leads to antibody production, organ destruction, and severe fibrosis in patients with ANCA-positive HP. A thorough understanding of the complex mechanisms of the disease might lead to pharmacologic strategies to inhibit the initiation and/or progression of ANCA-positive HP.

In conclusion, our case highlights that PR3-ANCA-positive HP can occur not only in the elderly but also in adolescents, and that early recognition and diagnosis is vitally important for appropriate treatment initiation to prevent irreversible organ damage and poor prognosis.

## Author contributions

**Conceptualization:** Kotaro Matsumoto.

**Supervision:** Nobuhiko Kajio, Kotaro Otomo, Kazuko Suzuki, Naoshi Nishina, Kento Kasuya, Naoki Oishi, Kaori Kameyama, Tsutomu Takeuchi.

**Writing – original draft:** Kotaro Matsumoto, Mitsuhiro Akiyama.
